# An Unusual Case of Müllerian Duct Cyst in an Adult Male: Radiological Diagnosis and Clinical Implications

**DOI:** 10.7759/cureus.87516

**Published:** 2025-07-08

**Authors:** Ujwala Bhanarkar, Shashank Durshetwar

**Affiliations:** 1 Department of Anatomy, All India Institute of Medical Sciences, Kalyani, IND; 2 Department of Radiology, Regional Referral Services Hospital, Amravati, IND

**Keywords:** computed tomography, congenital anomaly, cross-sectional imaging, cystic pelvic lesions, male pelvis, midline pelvic cyst, müllerian duct remnant cyst, paramesonephric duct, rectovesical space

## Abstract

Müllerian duct remnant cysts in males represent an exceedingly rare form of congenital anomaly resulting from incomplete regression of the paramesonephric (Müllerian) ducts during embryological development. We report a rare case of a 30-year-old male presenting with a history of intermittent pain in the right side of the abdomen and lower back for over eight months. There were no urological or gastrointestinal symptoms. A contrast-enhanced computed tomography (CECT) scan of the abdomen and pelvis revealed a well-defined, tubular, peripherally enhancing cystic lesion measuring 3.0 × 3.0 × 7.8 cm, located in the rectovesical space and extending down to the anal verge. The lesion demonstrated fluid attenuation, lacked septations or solid components, and showed no communication with adjacent organs. These features were highly suggestive of a Müllerian duct remnant cyst. The prostate was noted to be small for age, while other pelvic and abdominal organs were unremarkable. Given the unusual presentation and imaging features, this case adds to the limited literature on symptomatic Müllerian duct remnants in adult males. The report emphasises the importance of cross-sectional imaging in accurate diagnosis, differentiation from other midline pelvic cystic lesions, and guiding further management strategies, including the role of MRI and possible surgical intervention in symptomatic cases.

## Introduction

The development of the human genitourinary system is a highly orchestrated process that begins in early embryogenesis. In both sexes, the mesonephric (Wolffian) and paramesonephric (Müllerian) ducts coexist initially [1]. In genetic males (46, XY), differentiation into the male reproductive tract is initiated under the influence of testis-determining factor (TDF) and hormones secreted by Sertoli and Leydig cells. By the 7th week of gestation, Sertoli cells produce anti-Müllerian hormone (AMH), also known as Müllerian-inhibiting substance (MIS), which leads to regression of the Müllerian ducts [1]. In contrast, Leydig cells secrete testosterone, promoting the development of the Wolffian duct into male internal genital structures such as the epididymis, vas deferens, and seminal vesicles [2].

However, incomplete regression of the Müllerian ducts may occur due to inadequate AMH production or receptor defects. When regression is partial or asymmetric, remnants of the Müllerian ducts may persist, most commonly as Müllerian duct cysts, prostatic utricle cysts, or appendices testis [3]. Müllerian duct cysts are typically located in the midline, between the bladder and rectum, and are not connected to the urethra, unlike prostatic utricle cysts [4]. These cysts are usually lined by columnar or cuboidal epithelium and are non-communicating with the urogenital tract [5].

Müllerian duct remnant cysts are extremely rare in males and are often asymptomatic, discovered incidentally during pelvic imaging. When symptomatic, they may present with pelvic pain, urinary symptoms, constipation, infertility, or infection. Their diagnosis is primarily based on imaging modalities such as ultrasound, CT, and MRI, which help in delineating the cyst’s location, size, and relation to adjacent structures [6]. MRI, in particular, is valuable for its superior soft tissue contrast and multiplanar capabilities, allowing differentiation from other cystic pelvic masses such as seminal vesicle cysts, ejaculatory duct cysts, and enteric duplication cysts [7].

Due to their rarity, Müllerian duct cysts can pose diagnostic challenges and are often mistaken for other pelvic lesions, leading to inappropriate management. Histopathological confirmation following surgical excision remains the gold standard in unclear or symptomatic cases. This case report presents a classic example of a Müllerian duct remnant cyst in a 30-year-old male with chronic pelvic pain, highlighting the embryological background, radiological features, differential diagnosis, and clinical implications of this rare anomaly.

## Case presentation

A 30-year-old male presented to the outpatient department with complaints of intermittent pain on the right side of the abdomen and lower back, persisting for the past eight months. The pain was dull in nature, non-radiating, and not associated with bowel or bladder disturbances. There were no symptoms of fever, weight loss, vomiting, dysuria, hematuria, or altered bowel habits. The patient did not report any history of urinary tract infections, surgeries, or genitourinary anomalies during childhood. There was no significant family history of congenital disorders.

On clinical examination, the abdomen was soft and non-tender, with no palpable mass or organomegaly. Digital rectal examination was unremarkable. Basic blood investigations, including complete blood count, renal and liver function tests, and serum electrolytes, were within normal limits. Urinalysis was also unremarkable.

**Figure 1:**
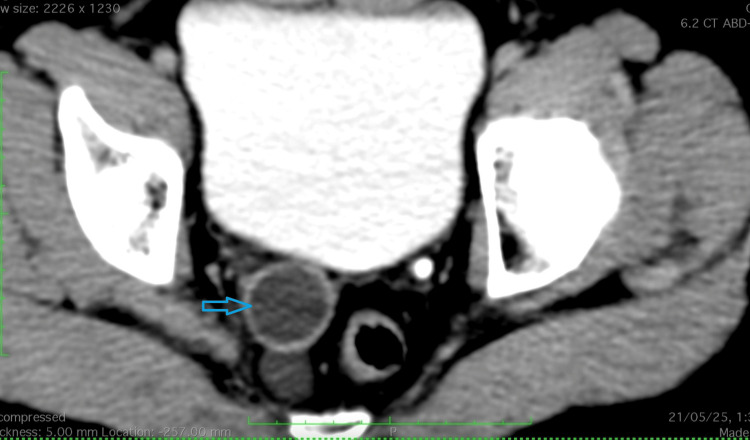
Contrast-enhanced computed tomography (CECT). Contrast-enhanced computed tomography (CECT) of the axial section of pelvis showing well-defined, tubular peripherally enhancing lesion in the rectovesical space, more towards the right paramedian region marked by an arrow.

**Figure 2:**
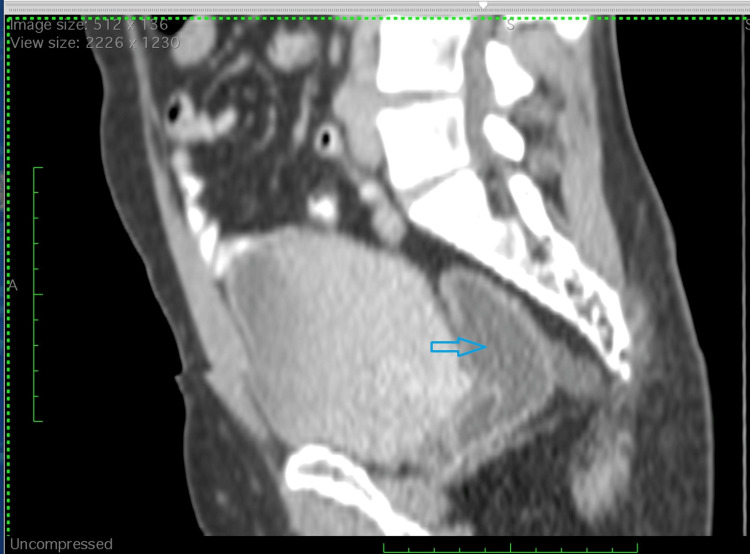
Contrast-enhanced computed tomography (CECT). Contrast-enhanced computed tomography (CECT) of the sagittal section of pelvis showing well defined, tubular peripherally enhancing lesion in the rectovesical space marked by arrow extending inferiorly toward the anal verge. The lesion demonstrates homogeneous fluid attenuation without definite solid components or septations. No communication with the bladder, prostate, or rectum is evident.

Given the persistence of pain without overt clinical findings, the patient was referred for imaging. A contrast-enhanced computed tomography (CT) scan of the abdomen and pelvis was performed. The CT images revealed a well-defined, tubular, peripherally enhancing cystic lesion measuring 3.0 × 3.0 × 7.8 cm, located in the rectovesical space, predominantly on the right paramedian side. The lesion was seen extending inferiorly from the level of the S1 vertebra, coursing anterior to the lower rectum and anal canal, and reaching down to the anal verge (Figures 1-2). Additionally, the inferior extent of the lesion appeared indistinct, which is acknowledged as a limitation of CT in soft tissue delineation. 

The cystic structure demonstrated homogeneous fluid attenuation, with no evidence of internal septations, calcifications, or solid enhancing components. There was no surrounding fat stranding, and no mesorectal or pararectal lymphadenopathy was noted. Importantly, the lesion did not show communication with the adjacent urinary bladder, prostate, rectum, or seminal vesicles. The prostate gland appeared slightly small for its age, while the urinary bladder, kidneys, liver, pancreas, spleen, and adrenal glands were normal. No radiopaque calculi or signs of obstruction were observed in the urinary tract. The bony structures were also normal, with no evidence of destructive lesions or degenerative changes. While definitive diagnosis requires histopathological confirmation or surgical excision, the radiologic and anatomical features in this case most closely align with a Müllerian duct remnant cyst.

Based on the location, morphology, and imaging characteristics, a diagnosis of a Müllerian duct remnant cyst was considered. The lesion was non-infected, non-communicating, and without any malignant features. The patient was advised to undergo further evaluation with pelvic MRI to better delineate soft tissue planes, confirm the absence of communication with the genitourinary system, and assist in treatment planning, but this was not performed due to logistical and financial constraints. At the time of reporting, surgical management was being considered due to the chronic nature of symptoms and the size of the lesion.

## Discussion

Müllerian duct remnant cysts in males represent a rare but clinically important anomaly of the male reproductive tract. The present case is unique in its size, anatomical extent, and clinical presentation, yet it shares similarities with several previously reported Müllerian duct cyst cases in terms of imaging features and embryological origin.

In a retrospective study by Shebel et al. (2013) evaluating lower male genitourinary tract cysts, Müllerian duct cysts were reported in 21% of cases. Most patients were asymptomatic, with cysts typically measuring less than 4 cm in maximum dimension [4]. In contrast, our case demonstrated a larger lesion (7.8 cm in craniocaudal length), extending from the S1 level to the anal verge, indicating a more extensive remnant.

In the study by Honore M et al. (2016), an MRI of the pelvis performed three days postoperatively revealed that the cystic structure adjacent to the prostate was a Müllerian duct cyst, which caused bladder outlet obstruction in a male patient with an HNF-1β gene deletion [5]. Unlike their patient, who presented with acute urinary retention and required urgent intervention, our patient had no urinary symptoms and no known genetic abnormalities.

In contrast to the case by He and Tang (2021) involving a 17-year-old male with lower abdominal pain and dysuria, our adult patient presented with nonspecific abdominal and back pain without urinary symptoms [7]. While both cases involved non-communicating rectovesical Müllerian duct cysts, the cyst in our case was larger and extended further caudally, emphasising the age-related variation in presentation and extent.

Compared to the case reported by Meng et al. (2012), where a giant Müllerian duct cyst presented as a perineal mass [8], our case involved a cyst located in the rectovesical space extending to the anal verge but not externally visible. While both were large cysts, Meng’s case was perineally protruding [8], whereas ours was entirely internal, highlighting differences in location and clinical visibility despite similar embryologic origins.

Another unique aspect of our case is the laterality. While Müllerian duct cysts are classically midline, our lesion was described as slightly right paramedian, a deviation occasionally seen in other reports, such as in a case discussed by Felderman T (1987), where mild deviation occurred due to mass effect on adjacent structures [3].

In contrast to the case reported by Jaidane et al. (2009), where an elderly male presented with acute urinary retention due to a giant Müllerian duct cyst [9], our patient was a younger adult with chronic abdominal and back pain and no urinary symptoms. While both cases involved large retrovesical cysts, the clinical presentation differed significantly, highlighting how the same anomaly can produce variable symptoms based on size, age, and anatomical impact [9].

To contextualise this case, it is compared with previously documented cases of Müllerian duct cysts in the literature, focusing on similarities and differences in patient demographics, clinical manifestations, imaging characteristics, and treatment approaches, as summarised in Table 1.

**Table 1:** Comparative summary of selected reported cases of Müllerian duct cysts in males including the present case.

Author (year)	Age/sex	Presentation	Size (cm)	Communication	MRI done	Management
Shebel et al. [4]	27-40 M	Mostly asymptomatic	<4	No	Yes	Observation
Honore M et al. [5]	24 M	Acute urinary retention, prostatic abscess	3-5	No	Yes	Transurethral drainage
He and Tang [7]	17 M	Scrotal mass of 6 years' duration	~5	No	Yes	Excision
Meng X et al. [8]	37 M	Mass in the perineum	50 × 40 × 30	No	Yes	Surgical excision
Present case	30 M	Right-sided abdominal/back pain	3 × 3 × 7.8	No	Advised	Surgical excision planned

## Conclusions

Müllerian duct remnant cysts are rare congenital anomalies in males that may remain asymptomatic or present with vague pelvic symptoms. This case highlights the need to include Müllerian duct cysts in the differential diagnosis of rectovesical pelvic lesions. Imaging, especially CT and MRI, is crucial for diagnosis and surgical planning. Early recognition and appropriate management can prevent complications and ensure optimal outcomes. This case adds to the growing, yet still limited, literature on Müllerian duct remnants and underscores the value of early imaging and individualised clinical decision-making in such rare entities.
